# Modulation of osteoclastogenesis by macrogeometrically designed hydrophilic dual acid-etched titanium surfaces

**DOI:** 10.1590/1807-3107bor-2024.vol38.0064

**Published:** 2024-07-15

**Authors:** Rainde Naiara Rezende de JESUS, Christos TSATSANIS, Camilla Christian Gomes MOURA, Darceny ZANETTA-BARBOSA, Andreas STAVROPOULOS

**Affiliations:** (a) Malmö University – MAU, Faculty of Odontology, Department of Periodontology, Malmö, Sweden.; (b) University of Crete – UOC, School of Medicine, Department of Clinical Chemistry, Heraklion, Crete, Greece.; (c) Universidade Federal de Uberlândia – UFU, School of Dentistry, Department of Endodontics, Uberlândia, MG, Brazil.; (d) Universidade Federal de Uberlândia – UFU, School of Dentistry, Department of Oral and Maxillofacial Surgery, Uberlândia, MG, Brazil.

**Keywords:** Dental Implants, Titanium, Hydrophobic and Hydrophilic Interactions, Osteoclasts, Gene Expression

## Abstract

The aim of this study was to evaluate the influence of implant macrodesign and surface hydrophilicity on osteoclast (OC) differentiation, activation, and survival *in vitro*. Titanium disks were produced with a sandblasted, dual acid-etched surface, with or without additional chemical modification for increasing hydrophilicity (SAE-HD and SAE, respectively) and different macrodesign comprising trapezoidal (HLX) or triangular threads (TMX). This study evaluated 7 groups in total, 4 of which were experimental: HLX/SAE-HD, HLX-SAE, TMX/SAE-HD, and TMX/SAE; and 3 control groups comprising OC differentiated on polystyrene plates (CCPC): a positive CCPC (+), a negative CCPC (–), and a lipopolysaccharide-stimulated assay positive control group, CCPC-LPS. Murine macrophage RAW264.7 cells were seeded on the disks, differentiated to OC (RAW-OC) by receptor activator of nuclear factor-κB ligand (RANKL) treatment and cultured for 5 days. Osteoclast differentiation and cell viability were respectively assessed by specific enzymatic Tartrate-Resistant Acid Phosphatase (TRAP) activity and MTT assays. Expression levels of various OC-related genes were measured at the mRNA level by quantitative polymerase chain reaction (qPCR). HLX/SAE-HD, TMX/SAE-HD, and HLX/SAE significantly suppressed OC differentiation when compared to CCPC (+). Cell viability was significantly increased in TMX/SAE and reduced in HLX/SAE-HD. In addition, the expression of Interleukin (IL)-6 and Tumour Necrosis Factor (TNF)-α was upregulated in TMX/SAE-HD compared to CCPC (+). Hydrophilic surfaces negatively modulate macrophage/osteoclast viability. Specifically, SAE-HD with double triangular threads increases the cellular pro-inflammatory status, while surface hydrophilicity and macrodesign do not seem to have a distinct impact on osteoclast differentiation, activation, or survival.

## Introduction

Peri-implant bone healing follows a thoroughly organized and sequential tissue repair process, primarily dependent on cellular cross-talk among macrophages (Mφ), osteoclasts (OCs), mesenchymal stem cells (MSCs) and osteoblasts (OBs), which finely couples the activities of bone resorption and new bone formation.^1^

Advances on oral implant technology, specifically modifications on macrodesign (e.g., thread design), surface topography and wettability have resulted in faster and superior quality of osseointegration, *i.e.*, bone-to-implant contact (%BIC).^2,3^ Specifically, thread design has an impact on implant primary stability and stress distribution at the bone-implant interface^4^ and thereby influences the amount/rate of osseointegration. For example, a trapezoidal and triangular thread-shaped design yields lower biomechanical stresses to the surrounding bone^5^, which metabolism allows normal bone and prevents bone necrosis; while square and trapezoidal cutting chambers decrease the maximum micro motion and accelerate and enhance %BIC through an intramembranous-like healing pathway.^6^

In regard to surface topography, moderately-rough, sandblasted, large grid, acid-etched (SLA) surface show faster osseointegration compared to machined surfaces^7^, while additional chemical treatment, rendering the surface hydrophilicity, promotes further enhanced host-implant interactions.^8,9^ Hydrophilic surface treatment influences the early healing process by upregulating the expression of angiogenic factors and anti-inflammatory mediators, and downregulating the expression of pro-inflammatory cytokines, thereby directing osteogenic differentiation and maturation of MSCs (*i.e.*, contact osteogenesis).^10^

More recently, evidence suggests that implant surface properties activate several components of the innate immune response following implant placement,^11^ which further modulate osteoclastogenesis in a surface dependent manner.^12,13^ Nevertheless, there is limited knowledge regarding the combined modulatory impact of implant macrogeometry and wettability on osteoclastogenesis. Thus, the present *in vitro* study aimed to evaluate the influence of implant thread design and surface hydrophilicity on OC differentiation, activation, and survival. Specifically, we hypothesized that a chemically modified micro-rough surface presenting significant hydrophilicity negatively modulates osteoclastogenesis, macrophage/osteoclast viability, activation and survival in comparison to the positive control group (*i.e.*, OC differentiated on polystyrene plates), not being influenced by the thread design.

## Methods

### Titanium disks and experimental groups

Disks (12 mm x 4 mm, Ø x L; Neodent^®^, Curitiba, Brazil) were of commercially pure titanium (CpTi) grade IV, exhibiting 2 different macrodesign, marketed as:

- Titamax^®^ (TMX): double triangular threads with similar grooves of 0.37 mm (Figure 1A).
  **Figure 1:**
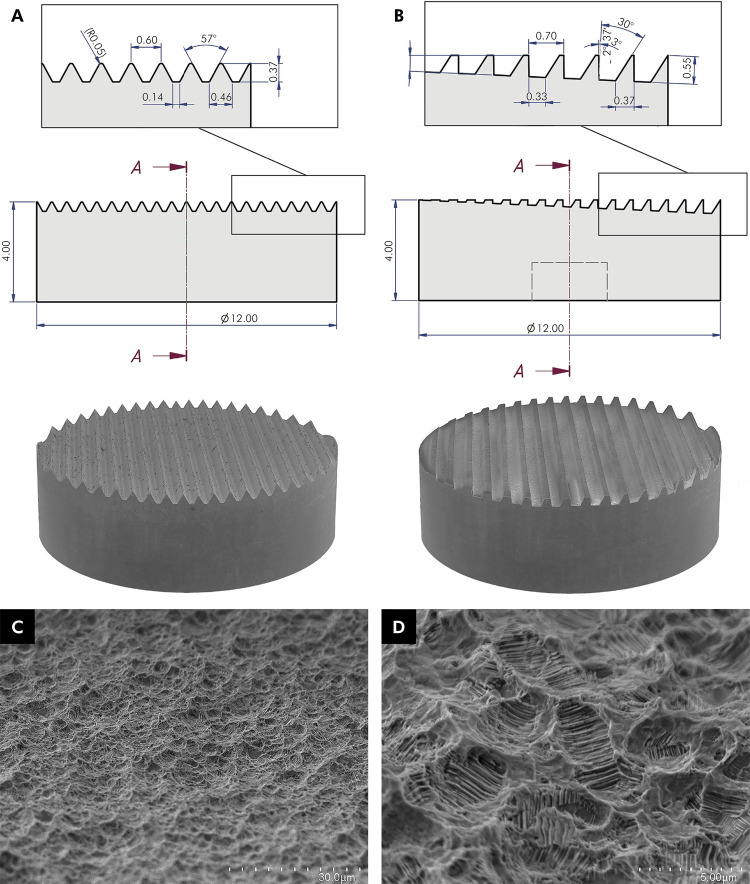
Schematic illustration and SEM micrographs of the threads’ geometric profile [shape, width, depth, pitch, face length (mm) and helix angle] and surface microtopography of the experimental disks. (A) Titamax® (TMX); (B) Helix® (HLX); (C-D) SEM micrographs of the surface microtopography following SAE treatment (1500x and 7000x magnification, respectively).
- Helix^®^ (HLX): dynamic progressive thread geometry with double threads varying between square and trapezoidal design to triangular threads exhibiting grooves of varied dimensions finishing in 0.55 mm (Figure 1B).

Further, each disk presented similar moderately rough microtopography, generated by means of sandblasting with 1.4–1.8 µm abrasive particles and dual acid-etching with hydrochloric (HCl) and sulfuric acid (H_2_SO_4_), but with varying surface wettability^8^:

- Acqua^™^ (SAE-HD): chemical modification resulting in significant hydrophilicity (contact angle < 5°; S_a_ = 1.26 µm).
- NeoPoros^®^ (SAE): no further chemical modification (contact angle > 90°; S_a_ = 1.44 µm).

Thus, this study evaluated 7 groups in total, 4 of which were experimental disks (HLX/SAE-HD, HLX/SAE, TMX/SAE-HD and TMX/SAE) and 3 control groups comprising OC differentiated on polystyrene plates (CCPC (+), CCPC (–) and CCPC-LPS), which are distinguished as follows:

- HLX/SAE-HD – hydrophilic surface with progressive thread geometry.
- HLX/SAE – hydrophobic surface with progressive thread geometry.
- TMX/SAE-HD – hydrophilic surface with triangular threads.
- TMX/SAE – hydrophobic surface with triangular threads.
- CCPC (+) – cells seeded directly on polystyrene-surface wells and cultured for 5 days in the presence of RANKL, as a positive control.
- CCPC (–) – cells seeded in the same fashion but cultured for only 12 h, without RANKL, as negative control.
- CCPC-LPS – cells treated with RANKL for 4 days and stimulated with 100 ng/ml lipopolysaccharide (LPS) for 24 h to secrete inflammatory cytokines, as an assay positive control group for MTT and gene expression.

### Mouse monocyte/macrophage cell line (RAW264.7) culture and differentiation to osteoclasts (RAW-OC) on titanium disks

RAW264.7, a murine monocyte/macrophage (Mo/Mφ)-like cell line (ATCC^®^ TIB-71^™^, LGC Standards GmbH, Wesel, Germany), was cultured at a density of 2 × 10^6^cells/ml in DMEM supplemented with 10% foetal bovine serum (FBS) and Penicillin-Streptomycin 10,000 µg/ml (Gibco^®^, Gaithersburg, USA) at 37°C, 5% CO_2_ and 95% relative humidity atmosphere. To differentiate RAW264.7 to osteoclasts, cells were seeded on the experimental disks (n = 3) placed in a 24-well plate at an initial population density of 3 × 10^5^/ml/well, subsequently treated with 100 ng/ml receptor activator of nuclear factor-κB ligand (RANKL; PeproTech^®^, Rocky Hill, USA) and cultured for 5 days, in triplicate and three independent experiments for each analysis. RANKL-containing medium was replaced after 3 days. The medium was removed after 5 days and cells were processed for further analysis.

### Specific enzymatic Tartrate-Resistant Acid Phosphatase (TRAP) activity

TRAP activity was measured using the Acid Phosphatase Colorimetric Assay Kit (Abcam^®^, Cambridge, UK), according to the manufacturer’s instructions. Readout of optical density at 405 nm was performed with a spectrophotometer. The final concentration of Acid Phosphatase (AP) was normalized to cell viability.

### 3-(4,5-Dimethylthiazol-2-yl)-2,5-Diphenyltetrazolium Bromide (MTT) assay

Cell viability of macrophage RAW264.7 cells and osteoclasts (RAW-OC) were assessed using the Vybrant^®^ MTT Cell Proliferation Colorimetric Assay Kit (Thermo Fisher Scientific^®^, Waltham, USA). The absorbance levels of each well were read at 570 nm using a spectrophotometer.

### Reverse transcriptase real-time quantitative polymerase chain reaction (RT-qPCR): 2-step qPCR sample preparation

The RT-qPCR method measured the mRNA levels of genes related to osteoclastogenesis, cell activity and survival after 5 days of differentiation. Extraction and purification of mRNA, and reverse transcription for relative quantification of gene expression employing real-time quantitative polymerase chain reaction (qPCR) were performed according to the protocol available as supplemental information. The gene expression levels of TRAP1, Cathepsin K (CTSK), Matrix Metalloproteinase (MMP)-9, Calcitonin Receptor (CALCR), Arginase (ARG)-1, Interleukin (IL)-6, Tumour Necrosis Factor (TNF)-α and Bcl-2-associated X protein (BAX) were investigated. The gene expression was quantified and fold regulation values were determined by normalizing cycle values (Ct) to glyceraldehyde 3-phosphate dehydrogenase (GAPDH) and beta-actin (ACTB; ΔCt) and again to the positive control [CCPC (+)] Ct (ΔΔCt).

### Confocal Laser Scanning Microscopy (CLSM) analysis

**Figure 2:**
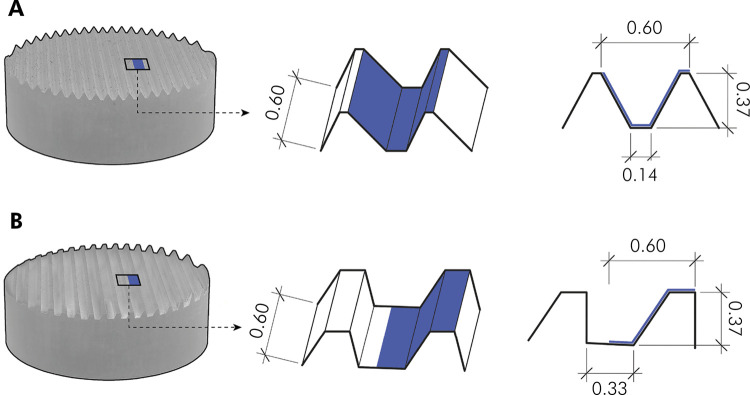
Schematic illustration of the region of interest (ROI) on the experimental disks to analyse osteoclast adhesion and morphology under SEM and CLSM. (A) Titamax® (TMX); (B) Helix® (HLX). Images of the ROI, equivalent to the thread pitch of each macrogeometry (grooves of 0.37 mm; blue area within the trapezoidal square), its three-dimensional and two-dimensional outlines.

Following cell fixation and permeabilization (Image-iT^™^, Invitrogen^®^, Carlsbad, USA), the actin cytoskeleton and cell nuclei were stained with phalloidin (0.1 µM/well; Alexa Fluor^™^ 488) and DAPI staining solutions (300 nm/well; FluoroPure^™^), respectively, according to the manufacturer’s instructions (Invitrogen^®^, Carlsbad, USA), and mounted in a small plexi (ProLong^™^ Gold Antifade Mountant). Confocal microscope images (TCS SP8 DLS^™^, Leica^®^, Wetzlar, Germany) were obtained in a region of interest (ROI) equivalent to the thread pitch of each macrogeometry (grooves of 0.37 mm), comprising a length and voxel depth of 0.60 x 0.60 x 0.37 mm (X x Y x Z; Figure 2A,B) at 25x magnification. Images were used for 3D assessment of cell area (µm^2^). 63x magnification (water immersion) micrographs were used for measurements of actin ring size (µm) and analysis of RAW-OC adhesion and morphology. Images were analysed using ImageJ 2.0 software (National Institutes of Health, Bethesda, USA).

### Scanning Electron Microscopy (SEM) analysis

Following cell fixation in Sørensen phosphate-buffered glutaraldehyde solution (4%, 0.1 M, pH 7.4), dehydration, and critical point drying (BAL-TEC CPD 030, BalTec Group, Pfäffikon, Switzerland), the disks were sputter-coated with gold/palladium (Polaron SC7640, Quorum Technologies Ltd, Kent, UK). Images at different magnifications (10x, 100x, 500x, 1500x and 3000x) were obtained under a SEM microscope (JSM-5600LV, JEOL^®^, Peabody, USA) operating in a low vacuum system with a tungsten filament electron source and polycarbonate filter at 20 kV. The RAW-OC morphology and adhesion were then analysed in a selected area as shown in Figure 2A,B.

### Statistical analysis

At least three independent experiments were performed for each analysis. Quantitative data were expressed as mean ± standard deviation (SD) and median. Initially, data were submitted to normality test (Shapiro-Wilk) and equal variance test (Levene). General linear statistical models were then applied for significant differences. MTT optical density and TRAP activity values were compared by one-way analysis of variance (ANOVA), followed by Turkey’s post hoc test for multiple comparisons with the value of statistical significance set at the 0.001 level. For the gene expression analysis, the aforementioned test was performed for comparison of TRAP1 mRNA levels, followed by Dunn’s post hoc test for comparisons with the positive control group (p < 0.05). Due to the asymmetrical distribution of data for gene expression levels of CTSK, MMP9, CALCR, ARG1, IL6, TNF and BAX, macrophage/osteoclast area and actin ring size, a Kruskal-Wallis non-parametric ANOVA was used, followed by Dunn’s post hoc test for comparisons with CCPC (+), and significance level was set at p < 0.05 and p < 0.001, respectively. The IBM SPSS Statistics software was used (IBM Company Inc., Chicago, USA).

## Results

### Osteoclast differentiation and cellular viability

**Figure 3:**
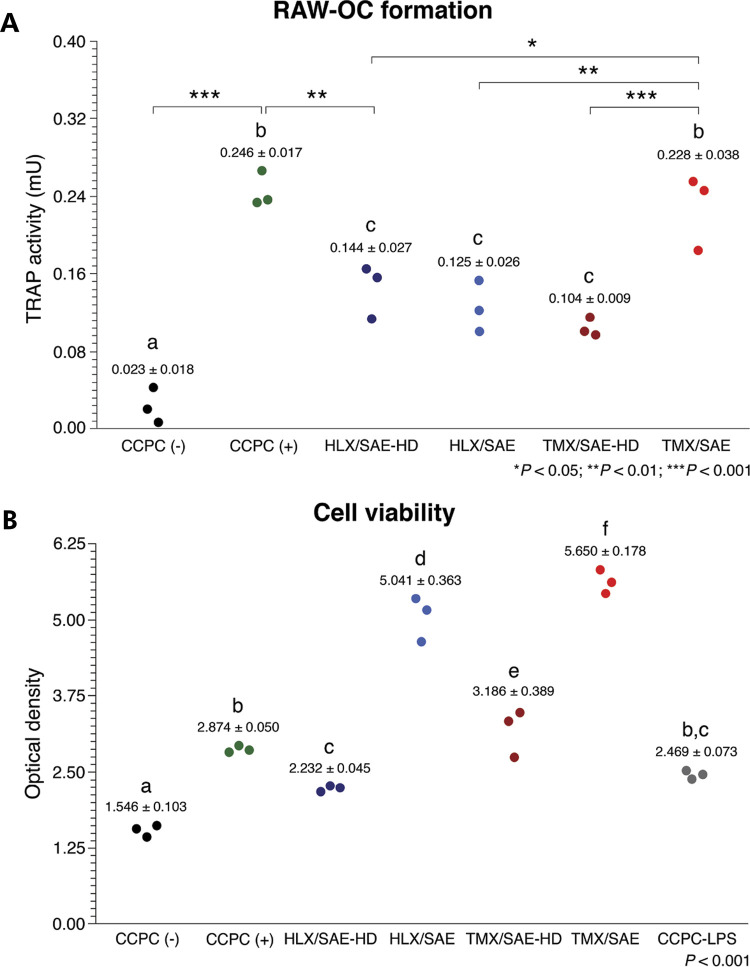
Mean ± s.d. and dot plots of induced osteoclast differentiation of macrophage RAW264.7 cells (RAW-OC formation) and cellular viability following 5 days of induced OC differentiation on experimental disks, compared to the control groups. (A) Specific enzymatic Tartrate-Resistant Acid Phosphatase (TRAP) activity assay; (B) MTT assay. Groups not sharing a letter are statistically significant at α = 0.05 (*), = 0.01 (**) and = 0.001 (***).

Following 5 days of RANKL-induced osteoclast differentiation, hydrophilic surfaces (HLX/SAE-HD and TMX/SAE-HD) significantly suppressed osteoclast differentiation (p < 0.01) when compared to the positive control group [CCPC (+)] (Figure 3A), partially confirming the alternative hypothesis. Similarly, HLX/SAE resulted in significantly reduced TRAP activity (p < 0.01); while TMX/SAE group did not have any influence on the modulation of OC differentiation when compared to CCPC (+) (p > 0.05).

The results of the MTT assay showed significantly different absorbance levels among the experimental groups (p < 0.001, Figure 3B); specifically, increased viability in TMX/SAE disks and reduced viability in HLX/SAE-HD disks were observed.

### Osteoclast-specific gene expression

**Figure 4:**
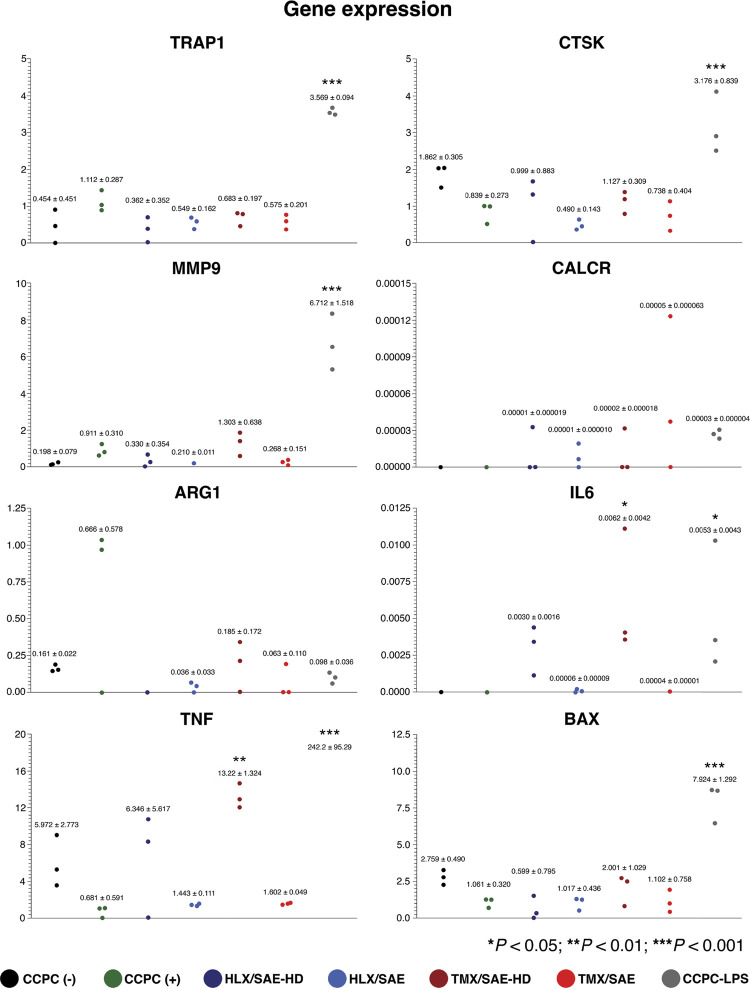
Mean ± s.d. and dot plots of the mRNA gene expression levels related to osteoclastogenesis and its negative regulation, osteoclast activity and survival following 5 days of induced OC differentiation on experimental disks, compared to the control groups. Significance level was set at α = 0.05 (*), = 0.01 (**) and = 0.001 (***).

None of the osteoclastogenesis and cell-survival related genes (TRAP1, CTSK, MMP9, CALCR, ARG1, and BAX) showed any statistically significant differences compared to the positive control group (p > 0.05; Figure 4). The only significant difference was in expression levels of genes associated with osteoclast-related inflammatory cytokines, when compared to CCPC (+). Specifically, expression of IL-6 and TNF was more than 6-fold and 19-fold upregulated in TMX/SAE-HD, respectively (p < 0.05 and p < 0.01; Figure 4).

### Qualitative image analysis of RAW-OC morphology and adhesion

**Figure 5:**
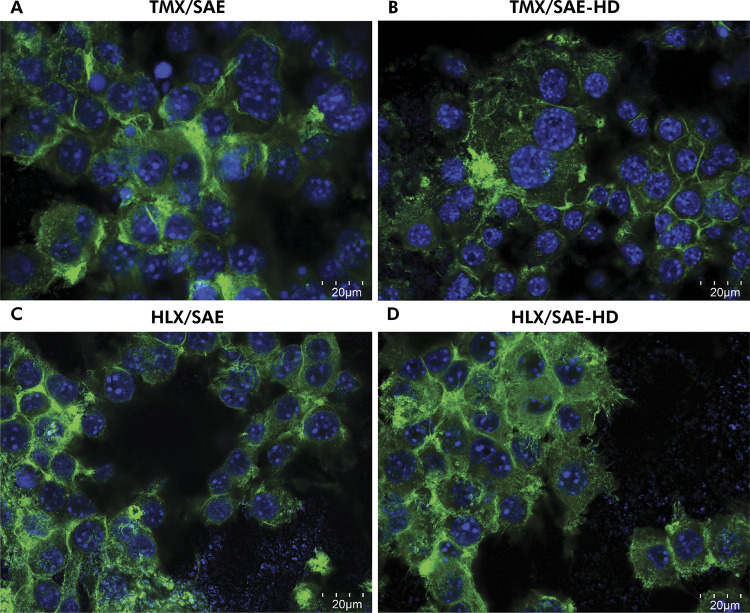
Qualitative image analysis of macrophage RAW264.7 cells and osteoclasts (RAW-OC) adhesion and morphology following 5 days of induced OC differentiation on titanium disks under the CLSM microscope (63x magnification), where cells were stained for actin cytoskeleton (*green*) and cell nuclei (*blue*). (A) TMX/SAE; (B) TMX/SAE-HD; (C) HLX/SAE; (D) HLX/SAE-HD. Cells cultured on all substrates formed extensive podosomes, actin-derived structures associated with integrin-mediated cell adhesion. Predominantly, larger actin rings were formed on hydrophobic SAE surfaces, whereas single podosomes as well as actin rings were mainly distributed along the cellular cytoplasm of hydrophilic SAE-HD.

Representative confocal micrographs demonstrate distinctive adhesion structures formed by multinucleated osteoclast-like cells and OCs on the experimental groups. Cells cultured on all substrates formed extensive podosomes, actin-derived structures associated with integrin-mediated cell adhesion. Predominantly, larger actin rings were formed on SAE surfaces (Figure 5A and 5C), whereas single podosomes as well as actin rings were mainly distributed along the cellular cytoplasm of SAE-HD (Figure 5B and 5D).

**Figure 6:**
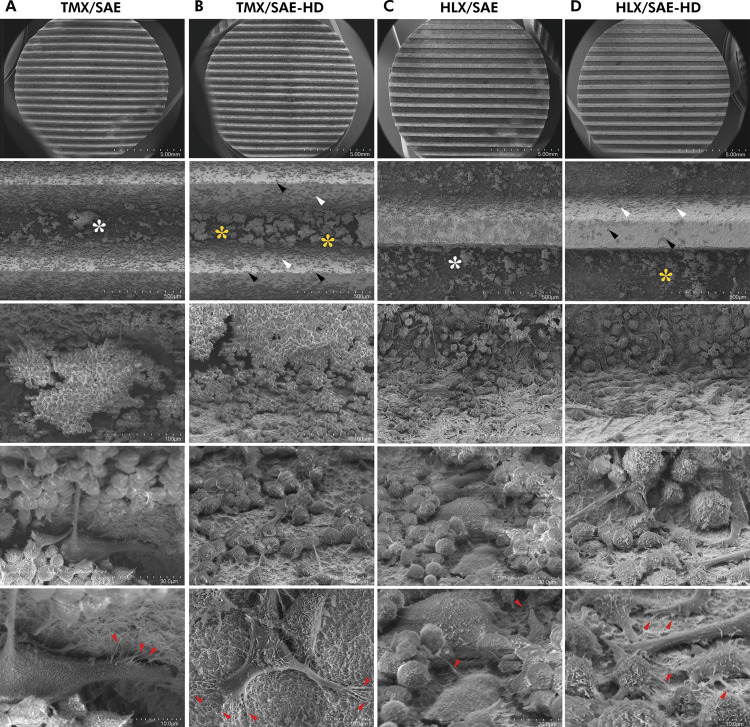
Qualitative image analysis of macrophage RAW264.7 cells and osteoclasts (RAW-OC) adhesion and morphology following 5 days of induced OC differentiation on titanium disks under the SEM microscope (10x, 100x, 500x, 1500x and 3000x magnification). (A) TMX/SAE; (B) TMX/SAE-HD; (C) HLX/SAE; (D) HLX/SAE-HD. Hydrophobic SAE surfaces revealed clusters of OCs along the entire pitch area, mainly in the valleys of the thread design (white asterisks). Hydrophilic SAE-HD surfaces showed cells randomly distributed in smaller clusters on top of the thread tips (black arrowheads) and flanks (white arrowheads) compared to hydrophobic SAE surfaces, while compact cell assembly were identified predominantly in the valley (yellow asterisks), regardless of the macrogeometry. Overall, SEM micrographs at 3000x magnification (bottom row) revealed actin-derived tightened structures and rod-like filopodia projections associated with integrin-mediated cell adhesion on both surface technologies (red arrowheads).

SEM images reveal cellular behaviour in terms of adhesion. In hydrophobic SAE surfaces, strictly aggregated clusters of RAW-OCs cells were identified along the entire pitch area, mainly in the valleys of both disks (Figure 6A and 6C). RAW-OC on hydrophilic surfaces were found in randomly distributed small clusters on top of the thread tips and flanks, while compact cell assembly were identified predominantly in the valley, regardless of the macrogeometry (Figure 6B and 6D). Overall, actin-derived tightened structures and rod-like filopodia projections associated with integrin-mediated cell adhesion were similar on both surface technologies, as observed in SEM micrographs at 3000x magnification.

### Quantitative image analysis of RAW-OC morphology and adhesion

**Figure 7:**
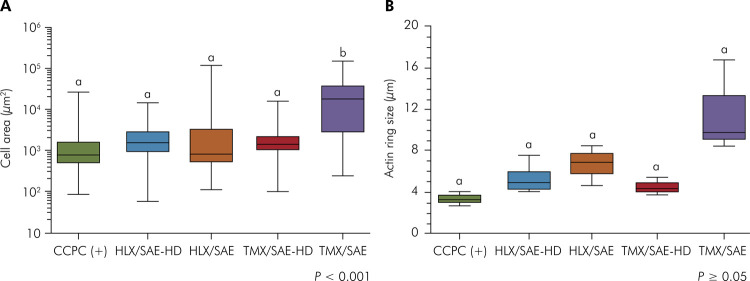
Box plots of the quantitative image analysis of macrophage RAW264.7 cells and osteoclasts (RAW-OC) adhesion following 5 days of induced OC differentiation on experimental disks, compared to the positive control group CCPC (+). (A) Cell area; (B) Actin ring size. Different letters indicate statistically significant differences at 0.001 (***).

RAW-OC cells on hydrophobic surfaces revealed the greatest range in area compared to hydrophilic disks and the control group (p < 000.1; Figure 7A). Significant differences were detected for TMX/SAE in comparison with the control group, HLX/SAE and SAE-HD surfaces. TMX/SAE group presented osteoclasts with the highest range in actin ring size (8.50–16.86 µm; median 9.75 µm), with no statistically significant differences compared to CCPC (+) (p ≥ 0.05; Figure 7B).

## Discussion

The aim of the present study was to investigate the influence of thread design and surface hydrophilicity on OC differentiation, activation, and survival in a murine macrophage-like cell line model. The hydrophilic (SAE-HD) and hydrophobic (SAE) surfaces investigated herein present similar surface topography. Nonetheless, Wennerberg et al.^14^ recently validated the structural formation of nanoparticles on SLActive surfaces manufactured in a similar way to SAE-HD herein, exhibiting nanotopographical features comparable to the cellular microenvironment.

Results obtained in the current study show that implant surface hydrophilicity negatively modulate osteoclast viability in the model of RANKL-induced osteoclastogenesis *in vitro*. Moreover, qualitative and quantitative assessment of cell morphology and adhesion revealed fundamental differences in a substrate-dependent manner. Integrin-based podosomes and actin rings, characterized in pre-mature and mature osteoclasts, respectively,^15,16^ were identified on all substrates. However, the total cell area of attached RAW-OC cells on hydrophilic surfaces was smaller than that measured on hydrophobic surfaces. Such a reduced cellular adhesion on chemically modified SAE-HD surfaces may have contributed to a decrease on cellular viability compared to SAE surfaces. Correspondingly, moderately rough hydrophilic surfaces were previously shown to decrease attachment of monocytes and negatively modulate osteoclast differentiation in an osteoclastogenesis-induced model of murine bone-marrow derived macrophages (BMMs) in comparison with hydrophobic surfaces.^12^

Recent *in vitro* studies have indicated that topographical modifications of biomaterials affect the assembly of the sealing zone (SZ) and resorption apparatus (RA). Particularly, surface roughness at the micro and nanoscale level was shown to limit SZ expansion throughout ridge-like barriers and to interfere with RA formation.^17^ These observations support a limited and short-term lasting formation of actin rings on structured micro- to nano-roughened substrates. Conversely, some studies support that osteoclastogenic differentiation and activation is equally increased by rough (R_a_ > 2 µm) and moderately-rough surfaces (R_a_ = 1-2 µm) as it occurs on bone, while these mechanisms are considerably decreased on smooth substrates displaying nearly absent actin rings.^18^

In spite of differences in cellular attachment and viability between hydrophilic and hydrophobic surfaces, the present findings confirm that enzymatic TRAP activity of cells grown and differentiated on HLX/SAE-HD, HLX/SAE, and TMX-SAE-HD surfaces was significantly lower than on TMX/SAE surfaces and CCPC (+). However, no significant differences were detected at the mRNA level of gene expression encoding this enzyme. Although increased synthesis of TRAP is an indicator of osteoclastogenesis, it is also highly expressed in fused and activated macrophages, playing a critical role in innate immune response.^19^ Recent data suggest that macrophage polarization into the pro-inflammatory phenotype (Mφ1) is primarily due to surface topographical modifications at the nanoscale level, rather than the biomaterial wettability, inhibiting osteoclast differentiation of its precursors.^20^ Henceforth, the absence of differences in the expression of genes encoding TRAP, in contrast to discrepancies found in the enzymatic activity, might own the formation of nanoparticles on SAE-HD and other aspects of cell adhesion and viability.

Considering that translation and protein abundance depend on cytoskeleton rearrangement, the main reason behind this finding is the presence of biochemical signals on bone, but not on biomaterials. Consistently, significantly higher TRAP activity is shown to be detected when in contact with osseous matrix compared to those grown on Ti surfaces.^18^ Hence, lack of significant differences at TRAP-related gene expression on Ti disks may be explained by the fact that functional membrane structures, such as SZ, RA, and functional secretory domain (FSD), are not entirely generated by mature OCs when cultured *in vitro* (*i.e.*, phenotypic change of gene expression by environmental influence).^21^ These changes in the cellular phenotype may result into disparities between gene expression and post-translational modification of protein levels.^22^ Furthermore, the correlation between mRNA and protein depends on other biological factors, such as cell cycle and its maturational stage, that may influence transcription levels, mRNA stability, translational rate, and protein turnover.^23^

During osteoclast activity, cell-matrix interactions occur including release of hydrogen ions (vacuolar-type H^+^-ATPase) in order to facilitate bone demineralization and organic matrix exposure through secretion of lysosomal proteolytic enzymes into the resorption lacunae.^24^ Gene expression levels did not reveal any significant effect of surface hydrophilicity and macrogeometry on osteoclast activation (CALCR) and phenotype associated with mineral and organic bone matrix cleavage and degradation (MMP-9 and CTSK, respectively), its negative regulator (ARG-1) nor on the expression of the pro-apoptotic gene BAX. Although there were no significant differences in the gene expression of CALCR gene, its upregulation in the TMX/SAE-HD and TMX/SAE groups may imply stimulation of a more mature OC phenotype compared to the positive control group.

Gene expression levels of IL-6 and TNF-α were significantly upregulated in TMX/SAE-HD. This event might suggest a relationship between a low rate of osteoclast differentiation accompanied by cytokine production and promotion of a pro-inflammatory microenvironment. In previous studies, however, hydrophilic surfaces were shown to activate the highest production of anti-inflammatory factors and down-regulate the expression of key pro-inflammatory cytokines (e.g., IL-1β, IL-6 and TNF-α) by osteoblasts (OB)^25^ and macrophage-like cells.^11^ Comparatively, the positive control group (cells differentiated on polystyrene-surface wells) revealed high level of ARG1 gene expression, suggesting prevalence of wound-healing macrophage phenotype (Mφ2) under RANKL-induced osteoclastogenesis when in the absence of an experimental Ti disk.^26^

Osteoclast differentiation of murine RAW 264.7 cells *in vitro*, comprehensively described in the literature, is possible through stimulation by RANKL for a minimum of 4 days, according to Lampiasi et al.^27^ The authors recently described the timing events and behaviour of OC differentiation of RANKL stimulation of this particular cell line. On the first 24 h, bipolar cells presented long filopodia among a few binucleated cells. Non-synchronous adhesion and fusion of lineage-committed mononuclear precursors increased following the second day through a so-called “fusopode bridge”, small membrane gaps and a cytoplasm mixing between the cells. This process led to large multinucleated cells, as observed more actively at 3 to 4 days, when active OCs were confirmed by positive staining after 4 days of RANKL-stimulated cells, supporting the present study methodology.

To date, this is the first study to evaluate osteoclast responses on hydrophilic substrates presenting different macrogeometry. Murine macrophage RAW264.7 cells have been extensively utilized in *in vitro* studies due to their expression of high levels of RANK and capacity to differentiate into OCs by treatment with RANKL.^28^ They are considered superior over the use of BMMs because of their purity, sensitivity to differentiation and prompt maturation, close correlation in gene expression and signalling, being functionally compared to primary isolated monocytes/macrophages.^22^ Nevertheless, results obtained herein shall be confirmed employing isolated BMMs in order to discriminate potential osteoclastogenesis-related processes against reported differences between immortalized macrophage cell line and primary macrophage-lineage cells (e.g., apoptosis/survival pathways and possible change in phenotype of subcultures). Further implications considering prospective animal studies and clinical outcomes grounded on laboratory-based evaluations should be entirely taken as assumptions to be validated.

## Conclusion

Chemically-modified hydrophilic surfaces with double triangular threads appear to negatively modulate macrophage/osteoclast viability and increase their pro-inflammatory status in a model of RANKL-induced osteoclastogenesis. Surface hydrophilicity and macrodesign do not seem to have a distinct impact on osteoclast differentiation, activation, or survival.
